# Metastasis to the colon from lung cancer presenting with severe hyponatremia and dyspnea in a young male: A case report and review of the literature

**DOI:** 10.3892/ol.2013.1208

**Published:** 2013-02-22

**Authors:** ALDO PEZZUTO, SALVATORE MARIOTTA, FEDERICA FIORETTI, STEFANIA UCCINI

**Affiliations:** 1 Cardiopulmonary Department, Sant’Andrea Hospital, Sapienza University, Rome I-00189, Italy; 2 Departments of Clinical and Molecular Medicine, Sant’Andrea Hospital, Sapienza University, Rome I-00189, Italy; 3 Pathology, Sant’Andrea Hospital, Sapienza University, Rome I-00189, Italy

**Keywords:** colon metastasis, colonoscopy, hyponatremia, immunohistochemical analysis, lung carcinoma

## Abstract

This study aimed to present the atypical clinical presentation and management of a metastatic lung cancer that had spread to an atypical location. Lung cancer is the most common cause of cancer-related mortality worldwide. The brain, liver, adrenal glands and bone are the most common sites of metastatic disease in patients with lung cancer. The reported incidence of symptomatic gastrointestinal metastases is 0.2–0.5%. Early diagnosis should be based on the observation of clinical symptoms and computed tomography (CT) imaging. In the present study, we describe the case of a 43-year-old male with a primary adenocarcinoma of the lung located in the lower right lobe. Following diagnosis, the patient underwent five lines of chemotherapy with a significant tumor reduction. Two years later, a mass located in the sigmoid colon was detected in the patient following a PET/CT scan. The clinical presentation was unusual with vomiting, headache, dyspnea and laboratory hyponatremia. A rare form of metastatic ulcerating adenocarcinoma was identified with colonoscopy, which was confirmed by immunohistochemical findings. A surgical approach was not performed due to the worsening condition of the patient. The patient demonstrated severe anemia and blood hypoxia, and one month later, the patient succumbed to disease. The metastasis may suggest an increase in tumor aggressiveness.

## Introduction

Lung cancer is the most common cause of cancer-related mortality worldwide. Half of the patients affected by lung cancer develop metastases, occurring more frequently in the lymph nodes, liver, bone, brain and adrenal glands (1). Gastrointestinal (GI) lung cancer metastases are extremely rare and the reported incidence is ∼0.5%, depending on the evaluation methods used, which include endoscopy, surgical specimens or autopsy (2).

Multidetector computed tomography (CT) with contrast medium is the optimum imaging procedure along with positron emission tomography (PET)/CT to detect colonic masses. The value of CT for depicting structural anatomy is widely appreciated and applied clinically to identify abnormalities in a non-invasive manner.

The most frequent clinical presentations of GI are abdominal pain and hemorrhage; however, other symptoms, including fatigue and dyspnea, are possible.

In the present study, we report the case of a young male who presented with weakness, dyspnea and headache prior to developing abdominal pain. These symptoms occurred following five lines of chemotherapy targeting the primary tumor. The clinical presentation was correlated with severe hyponatremia, most likely induced by the secretion of an ectopic antidiuretic hormone.

The main previously published studies reporting on GI metastases from the lung tumor are also reviewed. Written informed consent was obtained from the patient.

## Case report

### Clinical diagnosis and presentation

**Figure 1:**
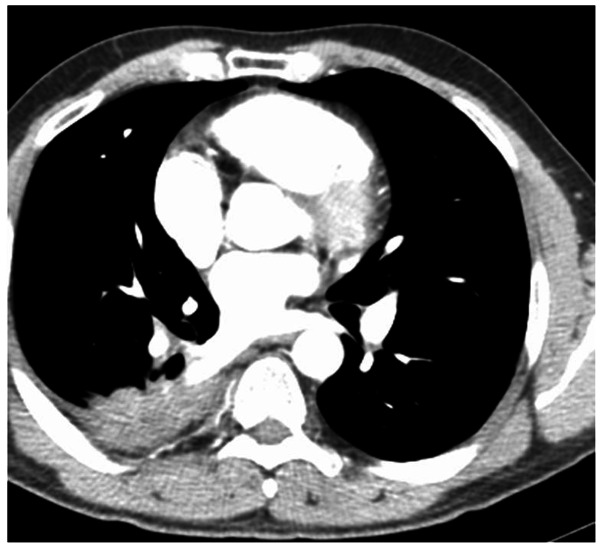
Total body computed tomography (CT) showing a lung mass located in the apical and postero-basal portion of the right lung.

A 43-year-old male with a 25-year smoking history (45 pack-years) was referred to the Cardiopulmonary Department in Sant’Andrea Hospital, Rome, Italy, in October 2009, due to a persistent cough and high temperature for several days. The parental history was positive for chronic obstructive pulmonary disease and cancer. The patient received antibiotic treatment without benefit over two weeks. Further investigations demonstrated mild anemia with a hemoglobin (Hb) concentration of 119 g/l and normal leukocyte and platelet counts. The oxy-hemoglobin saturation was also normal. A body CT scan revealed a lung mass measuring nearly 5 cm (Fig. 1) in the lower right lung and bronchoscopy with a bronchial lavage detected the presence of lung cancer cells. The patient underwent thoracotomy; however, it was not possible to remove the lesion due to infiltration of the main pulmonary artery. A diagnosis of adenocarcinoma involving vessels of the mediastinum was made, with evidence of neither lymph node nor distant metastases (T4N0M0, Stage IIIa). The patient underwent five lines of chemotherapy, beginning in December 2009: cisplatin + vinorelbine, three cycles; pemetrexed, six cycles; oral vinorelbine, three cycles; erlotinib for only three months due to the occurrence of an early skin side effect; gemcitabine, four cycles without significant changes in the natural course of the disease. Two years after the diagnosis, the patient was again admitted to our hospital as a result of fatigue, dyspnea at rest, headache and neuralgia of the lower leg. The hematological results were: Hb (11.0 g/l), platelet count (105×10^9^/l), white blood cells (12.1×10^9^ g/l), the sodium serum level was extremely low (120 mEq/l) with normal urinary and elevated D-dimer levels. The patient was hypoxemic with a resting PaO_2_ of 59 mmHg.

**Figure 2:**
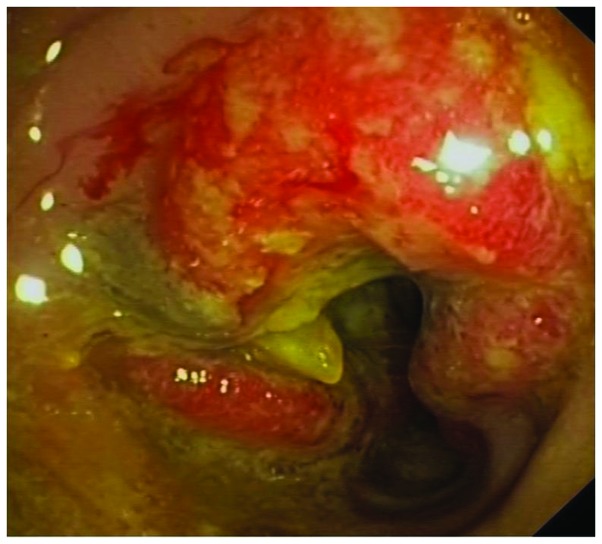
Endoscopic colonic ulcerating and bleeding lesion involving the sigmoid colon in all its extension. From this lesion, a biopsy for histology was obtained.

**Figure 3:**
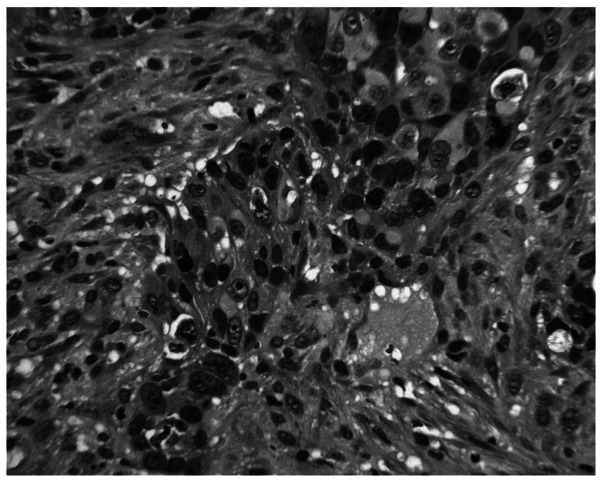
Histological sections revealed a colon metastatic adenocarcinoma demonstrating a poorly differentiated glandular pattern (hematoxylin and eosin staining, ×160).

**Figure 4:**
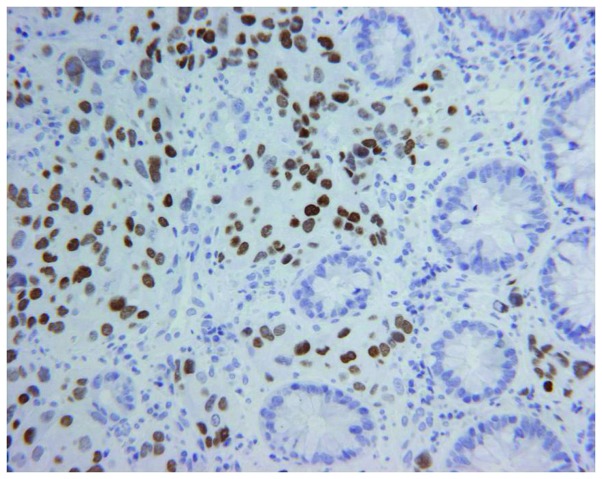
Intense nuclear staining for TTF-1 (peroxidase staining, ×63) and CK7 at immunohistochemistry of the neoplastic cells infiltrating the intestinal mucosa. The expression of CK20 and CDX2 was negative. TTF-1, transcription factor 1; CK, cytokeratin; CDX2, homeobox transcription factor 2.

A total body multidetected CT scan additionally revealed a single brain metastasis located in the left parietal lobe with concurrent GI involvement. A FDG-PET scan was also performed demonstrating a tracer uptake with high intensity in two sites; the lung and sigmoid colon. A colonoscopy was also performed showing a colonic ulcerating lesion which bled on touch (Fig. 2) and an endoscopic biopsy revealed neoplastic infiltration by a poorly differentiated adenocarcinoma of lung origin. Immunostaining detected a positive correlation between the neoplastic cells with thyroid transcription factor 1 (TTF-1) and cytokeratin 7 (CK7), with no evidence of CK20 and CDX2 expression (Figs. 3 and 4).

### Treatment

The patient underwent brain radiotherapy and treatment with dexamethasone, followed by moisturizing therapy. Although no obstructive symptoms were evident, a surgical treatment was recommended on the colonic lesion to avoid obstruction and perforation, however; it was not performed due to worsening of the patient’s general condition. One month later, the patient succumbed to severe lung failure, dehydration and metabolic disorder.

### Review of the literature

A search of the literature was conducted using Medline and Scopus databases. The first study that we examined identified 8,159 diagnosed cases of lung cancer collected between 1987 and 2008. The incidence of GI metastases in this study was 0.34%, 29 patients in absolute number. The specimens used to make the pathological findings were obtained either on surgical resection or on endoscopic biopsies (3). The data recorded included the stage of lung cancer at initial diagnosis, and the interval between diagnosis of lung cancer and the detection of GI metastasis. Only two of the 29 patients with GI metastases were identified as having colon metastatic presentation. In this study, all patients underwent contrast-enhanced abdominal CT and the most common clinical presentation was abdominal pain followed by anemia and jaundice. The most common histological type was squamous cell carcinoma, followed by adenocarcinoma. A perforation of the GI tract occurred in 22% of the patients. There were six small bowel metastases exhibiting either GI obstruction or perforation.

In the above-mentioned study, only 3 of the 21 lung cancer patients were diagnosed with colonic metastases, consisting of stomach or duodenal involvement. The most common symptoms were bleeding, hemorrhage and abdominal pain. The prognosis was poor upon the detection of metastases, particularly when they were atypical in nature. As is evident from a study of 8,493 new cases of lung cancer diagnosed between August 1998 and August 2007 (4), the incidence of GI presentation was 0.34% and among these patients, 29 of the 31 patients had stomach and small bowel involvement and only 2 demonstrated colonic metastases. The majority of the patients had synchronous metastases in other locations at the time of GI metastasis discovery, mainly in the lymph nodes, liver, adrenal glands, bone and brain. Another case of colonic metastasis from primary carcinoma of the lung was described by Hirasaki *et al*(5), who demonstrated a positive fecal occult blood test. The histological type was squamous (5).

## Discussion

The case described in the present study is characterized by an atypical location of metastasis from lung cancer with an atypical course of disease and clinical presentation. The management of metastasis from lung cancer with atypical symptoms is the focus of this study. These symptoms are mainly due to the ectopic production of antidiuretic hormones by tumors. The observation of metastases in the large bowel, although rare, have to be considered in cases where the lung cancer is not well controlled and the patient does not exhibit a good response to chemotherapy. The initial stage and smoking history of the patient may affect the course of the disease.

The imaging techniques most commonly used for the detection of primary and metastatic lesions are the multi-detecter CT scan and PET-CT imaging, which are useful in the early detection of tumor lesions. The typical clinical presentations of GI metastases were variable; the majority of the patients with symptomatic gastric and/or duodenal metastases demonstrated abdominal pain (6,7), GI bleeding, obstruction and intestinal perforation.

The circulating tumor cells are important in the spread of neoplastic lesions to other organs (6), although metastases from primary lung carcinoma in the small bowel are more frequently detected than those in the large bowel. In this regard, Nishizawa *et al* have recently described seven cases (0.17%) of symptomatic small bowel metastases from 4,114 patients with lung cancer referred to the author’s institution between 1995 and 2005 (8). The median life expectancy following detection was 6 months, and the study revealed that a surgical approach is not always feasible due to increased perioperative risks. Large bowel metastasis was not described in this study.

The spread of tumor cells affects prognosis. The diffusion of disease by the hematic or lymphatic routes causes a worsening of clinical symptoms and survival rate (9). The direct invasion of the esophagus is more likely than bowel metastasis via the hematic route as the bowel is an organ which is distant from the bronchus (10,11). We hypothesize that its rare presentation is due to arterial perfusion of the left large bowel that is provided by the mesenteric inferior artery. This artery originates from the abdominal aorta at the level of the L3 vertebral body, and one of its branches is the sigmoid artery which supplies the descending colon, sigmoid colon and superior part of the rectum.

In the present study, we advance the hypothesis that the burden of disease is due to the aggressiveness of the tumor, and in addition, due to young age, the tumor cells propagate extremely fast and reach the arterial system of the colon.

The patient initially presented with dyspnea at rest, nausea and neuralgia of lower leg, most likely due to compression of the femoral nerve roots and also fatigue. However, neither rectal bleeding nor obstruction was observed.

An important consideration is that hyponatremia syndrome has a wide range of symptoms from nausea and vomiting to asthenia and edema of the brain, depending on the levels of sodium. In the metastatic phase, these symptoms are caused by low serum sodium levels, which form a metabolic condition in which there is not enough sodium in the body fluids outside of the cells (12). It is a common electrolyte abnormality observed frequently in cancer patients. It may be caused by the inappropriate secretion of antidiuretic hormone (13).

In this study, we identified that poorly differentiated adenocarcinoma was also atypical. A study by Berger *et al*(7) reported that squamous cell lung carcinoma causes small bowel metastases more frequently than other histotypes. An additional consideration is that the choice of chemotherapy for first and second line treatment is between classical and personalized treatment options, with human epidermal growth factor receptor tyrosine-kinase inhibitors (14). When the therapy is ineffective and the disease continues to advance, only best supportive care can be used.
